# An assessment of the clinical relevance of coracoid graft osteolysis following the Latarjet procedure: a clinical and radiological review

**DOI:** 10.1016/j.jseint.2024.03.004

**Published:** 2024-03-28

**Authors:** Ryan S. Ting, Bob Jang, Nicholas Murray, Tiffany G. Williams, Isabella L. Kang, Yon Su, Tam Anh Nguyen, William E. Ridley, Blake R. Manowski, Michelle Caudwell, Linda Martin, John N. Trantalis

**Affiliations:** a Orthocentre Orthopaedic Research Institute, Sydney, NSW, Australia; b Faculty of Medicine, University of New South Wales, Sydney, NSW, Australia; c Department of Orthopaedic Surgery, Concord Repatriation General Hospital, Sydney, NSW, Australia; d Faculty of Medicine, University of Sydney, Sydney, NSW, Australia

**Keywords:** Latarjet, Osteolysis, Resorption, Coracoid, Bone block, Instability, Shoulder, Dislocation

## Abstract

**Background:**

The Latarjet procedure was developed for the treatment of anterior shoulder instability in young, high-demand patients with attritional glenoid bone loss, whose risk of redislocation following primary dislocation may exceed 90%. Coracoid graft osteolysis and prominent screws are commonly observed in late computed tomography (CT) scans of patients who re-present following the procedure, but the clinical relevance of osteolysis in the overall Latarjet cohort is undetermined. We aimed to evaluate clinical and radiological outcomes in patients who underwent the Latarjet procedure, and to determine if severe coracoid graft osteolysis compromised clinical outcomes.

**Methods:**

This was a retrospective analysis of patients who underwent the open Latarjet procedure. Patients were invited via an e-questionnaire that contained a Western Ontario Shoulder Instability Index (WOSI), and queried about redislocation and reoperation since index surgery. Preoperative glenoid bone loss was calculated on CT using the best-fit circle method. Osteolysis was graded (0, screw head buried in graft; 1, screw head exposed; 2, threads exposed; 3, complete resorption/severe osteolysis) at the level of the proximal and distal screws respectively, on axial CT scans performed ≥ 12 months postoperatively.

**Results:**

Between 2011 and 2022, a single surgeon performed 442 Latarjet procedures. One hundred fifty eight patients responded to the questionnaire at median (interquartile range [IQR]) 44 (27-70) months postoperatively, among whom the median (IQR) WOSI score was 352 (142-666) points (0 = best, 2100 = worst). Recurrent anterior instability occurred in 3/158 (2%) patients. One patient required reoperation for this indication. Among patients who had CT scans ≥ 12 months postoperatively (median [IQR] 40 [29-69] months), 1 patient developed severe osteolysis around both screws (WOSI = 90), 17/62 (27%) patients developed severe osteolysis around 1 screw, all of which were proximal (median [IQR] WOSI = 235 [135-644]), and 44/62 (71%) patients did not develop severe osteolysis around either screw (median [IQR] WOSI = 487 [177-815]). There were no statistically significant differences in WOSI scores between groups based on the presence of severe osteolysis.

**Conclusion:**

The Latarjet is reliable procedure that has a low rate of redislocation and reoperation. Severe coracoid graft osteolysis occurs with time, and always affects the proximal graft first. The presence of severe osteolysis did not compromise clinical outcomes.

The open Latarjet procedure is a well-established treatment for traumatic anterior shoulder instability. Up to 92% of young, active patients redislocate their shoulders within 14 months of their initial dislocation.^20^ Despite the procedure becoming increasingly common, one reservation about the Latarjet procedure is its complication rate, which review articles have reported may occur in up to 15% of cases.^8^

Coracoid graft osteolysis is a frequently cited complication.^4^ Zhu et al^21^ proposed a 4-point system to grade the amount of osteolysis at the level of each screw on an axial computed tomography (CT) view in 57 open Latarjet patients, and included the worst osteolysis in each shoulder for analysis. They found no statistical differences in range of motion or Constant-Murley scores at final follow-up between the 4 groups based on the grade (0-3) of osteolysis.^21^

Bones adapt to the degree of mechanical loading in accordance with Wolff’s Law.^9^ Therefore, the transfer of the coracoid to a nonanatomic position and the subsequent changes in loading mean that some degree of coracoid graft osteolysis is likely. A potential clinical consequence of coracoid graft osteolysis is anterior shoulder pain, which has been attributed to irritation of the subscapularis by prominent screws.^10^^,^^19^

Therefore, we hypothesized that with time, the majority of Latarjet patients, not just those who re-presented due to unexplained anterior shoulder pain, would have some degree of screw prominence secondary to coracoid graft osteolysis on postoperative CT scan. The robust clinical outcomes of the Latarjet procedure, however, suggest that osteolysis does not compromise clinical outcomes. The aims of the present study, therefore, were to evaluate clinical and radiological outcomes in patients who underwent the open Latarjet procedure, and to determine if the severity of coracoid graft osteolysis was associated with patient-rated outcomes.

## Materials and methods

This was a retrospective cohort study. This study was conducted upon acquisition of ethics approval from the Sydney Local Health District Human Research Ethics Committee (Reference Number: 2022/ETH01561) and the Ramsay Research Governance Office (Reference Number: 2021/ETH/1018).

### Inclusion and exclusion criteria

Patients who underwent an open Latarjet procedure performed by a single surgeon (J.T.) were eligible for inclusion. Patients with concomitant rotator cuff tears or superior labral anterior-posterior tears that required repair were excluded. Patients who had less than 6 months of clinical follow-up were excluded.

### Preoperative workup

After a full history and examination, patients were sent for magnetic resonance arthrography including scans with the shoulder in abduction and external rotation, and also axial CT scans of both shoulders, with 3-dimensional reconstructions of the glenoid with the humeral head subtracted and vice versa.

### Surgical technique

The Latarjet procedure was indicated for recurrent traumatic anterior shoulder instability that caused functional limitations, or for first-time dislocators, those who were playing professional contact sports. Patients were placed in the semi-reclined position. The Latarjet was performed under a general anesthetic with an interscalene block. A medial skin incision and a deltopectoral approach were used to identify the conjoined tendon, which was traced proximally to identify the tip of the coracoid process. The pectoralis minor was first detached from the medial side of the coracoid, and then the periosteum elevated off the inferior surface. The coracoacromial ligament was then detached from the lateral aspect of the coracoid. The coracoid was osteotomized just anterior to the coracoclavicular ligaments using a 90° oscillating saw (DYONICS Power Oscillating Saw Interface; Smith & Nephew, Watford, England), and sometimes completed laterally with a curved osteotome to avoid fracturing into the glenoid. Hemostasis was achieved at the osteotomy site by application of bioresorbable Bone Wax (ETHICON, Raritan, NJ, USA). The coracoid graft was freed of its remaining soft tissue attachments, sparing the insertion of the conjoined tendon.

Depending on the amount of bone loss and the shape of the coracoid, the inferior (traditional technique) or medial (congruent arc technique) surface of the coracoid graft was prepared to create a flat bleeding surface using an oscillating saw. Two 4-mm drill holes were created using a Coracoid Drill Guide (Arthrex, Naples, FL, USA). The coracoid graft was then positioned over the prepared anterior glenoid neck such that a congruent joint surface was created. Two 1.6-mm Guide Wires (Arthrex, Naples, FL, USA) were advanced through a Parallel Drill Guide (Arthrex, Naples, FL, USA). The drill guide was removed, and the appropriate screw length was measured over the guidewire. The proximal cortices were drilled using a 2.75-mm cannulated drill bit to create a lag screw effect. Two 3.75-mm fully threaded, cannulated, self-tapping screws with washers were then inserted over the guidewire. Occasionally, solid titanium screws with washers were used if the bone seemed extraordinarily hard (eg, professional athletes).

Two 1.8-mm FiberTak (Arthrex, Naples, FL, USA) suture anchors were placed in the anteroinferior glenoid rim, and repaired to the capsule, ensuring that the coracoid graft remained extracapsular. The wound was closed and dressed per standard techniques, with the occasional use of a drain.

### Postoperative care

Patients were immobilized in a sling (SlingShot 3 Shoulder Brace; Breg, Inc., Carlsbad, CA, USA) for the first 4 weeks postoperatively. Patients were encouraged to perform hand and elbow range of motion exercises without resistance and were limited to shoulder shrugs for the first 6 weeks. Between 4 and 6 weeks, they only had to wear the sling outside of the home.

From 6 weeks to 12 weeks postoperatively, patients were commenced on a graded exercise program progressed from active shoulder movements to isometric exercises with resistance bands. The carrying restriction was 2 kg at 6 weeks, and increased by 1 kg per postoperative week. Patients were allowed to recommence weight training in the gym from 12 weeks onwards, after a clinical review. Patients were referred for an X-ray at 6 weeks, and a CT scan at 12 weeks postoperatively for standard evaluation.

### Data collection

We identified all patients who underwent a Latarjet procedure by screening our practice management software using the respective item codes. The list of Latarjet patients were then contacted via e-mail and/or telephone to participate in the study. Patients who consented to participate had their demographic variables (date of birth, sex, insurance status, and previous surgical history) extracted from electronic medical records. Preoperative CT scans were reviewed, and the amount of attritional glenoid bone loss was calculated using the best fit circle method described by Sugaya et al.^17^ In addition, they completed an electronic questionnaire that contained the Western Ontario Shoulder Instability Index (WOSI), and queried if they had redislocated their shoulders since index surgery, if they had undergone any operations on their shoulder prior to index surgery, and if they had undergone any subsequent operations on their shoulder since index surgery. In the questionnaire, patients were offered a shoulder CT scan. If a patient already had a previous shoulder CT scan performed ≥ 12 months postoperatively, then the latest scan was used to evaluate coracoid graft osteolysis.

### Grading of osteolysis

Coracoid graft osteolysis was graded on axial CT at the level of both the proximal and distal screws using the 4-point system developed by Zhu et al^21^ (Grade 0, screw head buried in coracoid graft; Grade I, only screw head is exposed; Grade 2, screw shaft partially exposed, with residual bone on glenoid neck; Grade 3, screw head and shaft are totally absorbed, with no residual bone on glenoid neck). Only scans that were performed ≥ 12 months after index surgery were analyzed for their association with patient WOSI scores.

### Statistical analysis

Quantitative variables with skewed distributions were reported as median (quartile 1-quartile 3), and compared between groups using nonparametric Mann-Whitney *U* tests. *P* < .05 was considered statistically significant. The median WOSI scores were plotted on a histogram with a reference line at the patient acceptable symptomatic state (PASS) of 571 points or less based on a previous study investigating the PASS following the Latarjet procedure.^13^ Bivariate Spearman correlation tests were conducted to determine the strength of the association between postoperative WOSI scores and the collected demographic variables, preoperative glenoid bone loss, postoperative coracoid graft osteolysis, and whether they had a redislocation post-Latarjet. All analyses were performed using SPSS Version 26 (IBM Corp., Armonk, NY, USA).

## Results

### Patient selection

**Figure 1:**
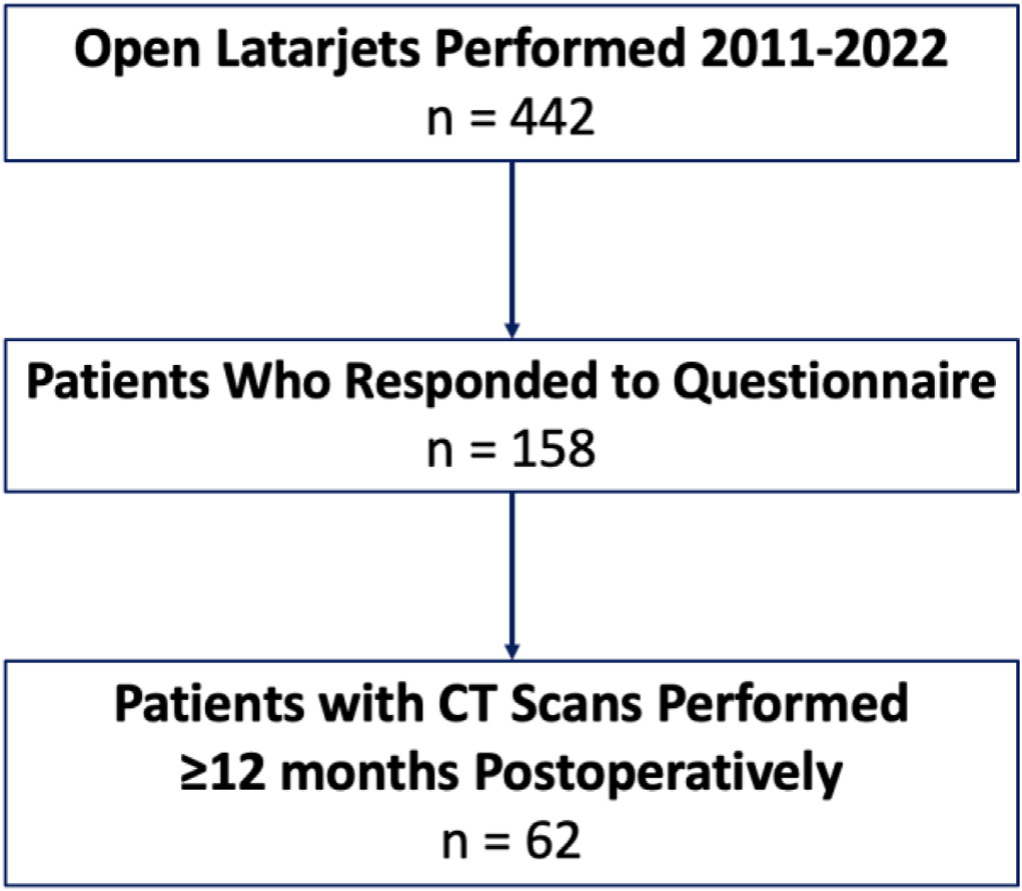
Patient selection flowchart. *CT*, computed tomography.

From January 1, 2010 and March 1, 2022, a single surgeon (J.T.) performed 442 open Latarjet procedures. One hundred fifty eight Latarjet patients accepted the study invitation and answered the electronic questionnaire. A further 62 patients either had a pre-existing CT scan or proceeded to undergo a new CT scan that was performed ≥ 12 months after index surgery (Fig. 1).

### Patient characteristics

Of the 158 patients who responded to the questionnaire, the median (interquartile range [IQR]) age at the time of surgery was 24 (19-30) years, 135/158 (85%) of patients were male, and 142/158 (90%) were privately insured, and the median (IQR) amount of attritional glenoid bone loss present on preoperative CT was 15% (5%-20%) (Table I).

**Table I:** Descriptive characteristics of the study population.

Overall patient characteristics	
Age, median (IQR), y	24 (19-30)
Sex, males, no. (%)	135/158 (85%)
Had surgery on ipsilateral shoulder pre-Latarjet, no. (%)	31/158 (20%)
Insurance type	
Private, no. (%)	142/158 (90%)
Self-funded, no. (%)	7/158 (4%)
Worker’s compensation, no. (%)	8/158 (5%)
Public, no. (%)	1/158 (1%)
Professional collision-sport athletes, no. (%)	12/158 (8%)
Preoperative glenoid bone loss, median (IQR), %	15% (5%-20%)

*IQR*, interquartile range.

### Outcomes

The median (IQR) time to final follow-up was 44 (26-29) months for patient-rated outcomes, and 40 (29-69) months for CT scans that were performed ≥ 12 months postoperatively. At final follow-up, the median (IQR) WOSI score was 339 (138-648) points. Of the 158 included patients, 4 experienced dislocations of the same shoulder post-Latarjet. However, one of these was a posterior dislocation. Three patients underwent subsequent surgery on the same shoulder post-Latarjet. One was a revision Latarjet procedure, 1 was for arthroscopic removal of loose bodies, and 1 was a posterior capsulolabral repair. One broken screw was noted on the postoperative scans of 3 patients. There were no infections, fractures, or nonunions. There were 2 transient, but no permanent neurological complications, fractures, or nonunion.

Older patients were more likely to achieve a higher WOSI score (r = 0.183, *P* = .022), whereas those who were self-funded were more likely to have lower WOSI scores (r = −0.158, *P* = .049). Sex, having undergone previous stabilization procedure(s), insurance type, or the amount of glenoid bone loss preoperatively did not correlate with WOSI scores (Supplement File 1).

### Coracoid graft osteolysis and WOSI

**Figure 2:**
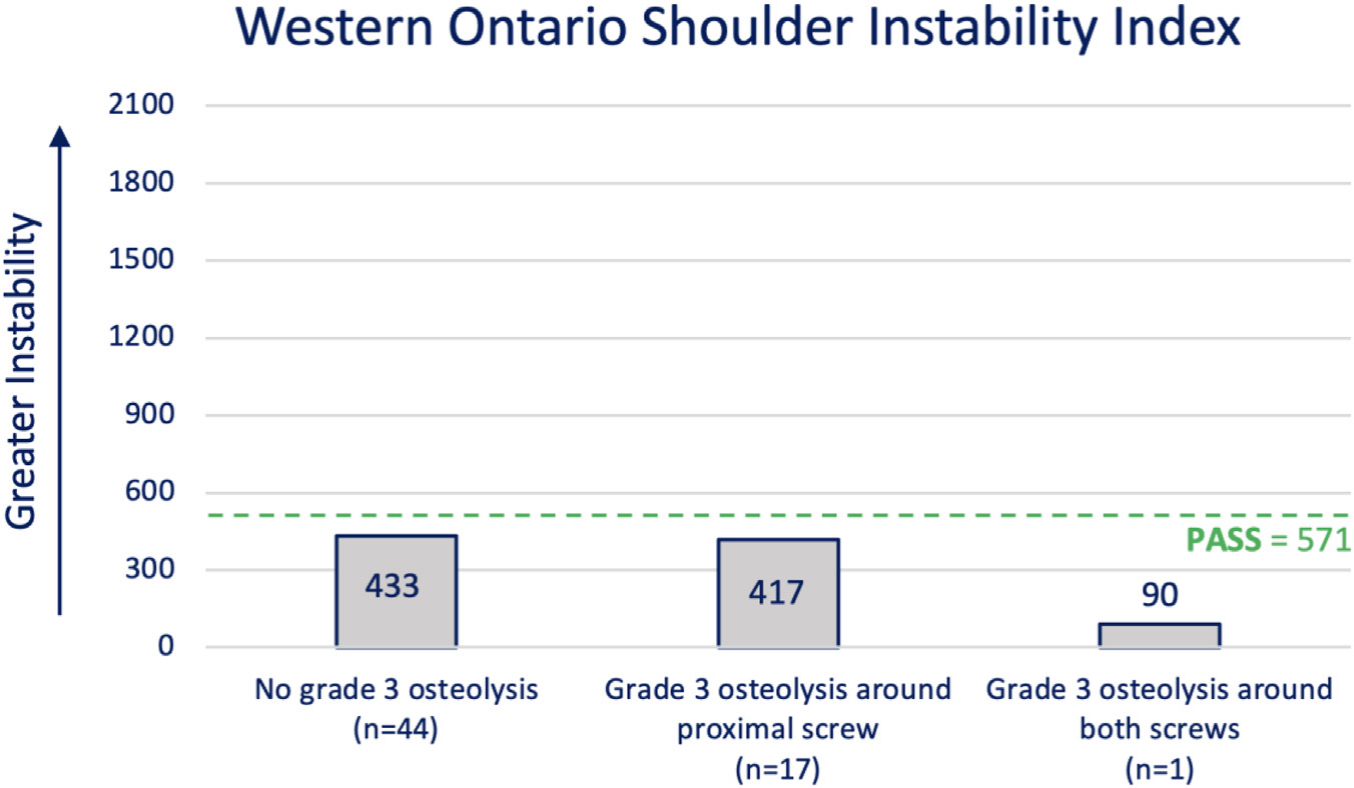
Median WOSI, grouped by the presence of severe osteolysis on CT scans performed ≥ 12 months postoperatively. *PASS*, patient acceptable symptomatic state; *CT*, computed tomography; *WOSI*, Western Ontario Shoulder Instability Index.

Of the patients who had a CT scan performed ≥ 12 months postoperatively, 44/62 (71%) patients did not have Grade 3 osteolysis around either screw, 17/62 (27%) of patients had Grade 3 osteolysis around one screw—all of which occurred around the proximal screw, and 1 patient had Grade 3 osteolysis around both screws. There were no statistical differences in WOSI scores between patients who had no Grade 3 osteolysis around either screw, patients who had Grade 3 osteolysis around one screw, and the one patient who had Grade 3 osteolysis around both screws. The median WOSI of each group was below the PASS of 571 points or less (Fig. 2).^13^ That is, these patients had an acceptable symptomatic state, as a low score implies a better functioning shoulder.

## Discussion

The major finding of the present study was that although severe coracoid graft osteolysis may develop around at least 1 screw in 29% (18/62) of patients 3 years post-Latarjet, severe osteolysis was not associated with poorer clinical outcomes, affirming the hypothesis. The present study found that the rate of recurrent anterior shoulder instability was 1.9% following the Latarjet procedure, and that the revision rate for this indication was 0.6%.

The prevalence of severe coracoid graft osteolysis in the present study was nearly 3-fold the 6/57 (11%) reported by Zhu et al.^21^ However, this was unsurprising as our CT scans were performed at median 40 months postoperatively compared to the 12-month scans performed by Zhu et al.^21^ Furthermore, Zhu et al^21^ only evaluated osteolysis at the level of the proximal screw, whereas our study evaluated osteolysis at both the proximal and distal screws. Nonetheless, this did not contribute to the higher rate of osteolysis in our cohort as severe osteolysis, where present, uniformly affected the proximal screw.

Di Giacomo et al^7^ divided the coracoid graft into 8 parts on sagittal CT views to evaluate osteolysis within each segment in a series of 26 open Latarjet patients. Although the Di Giacomo system is a more thorough method of quantifying the amount of coracoid graft osteolysis, we opted to use the Zhu classification as it is a straightforward, highly reliable system that requires only axial CT views, which in our setting are the default views produced by radiology providers. They found that the most superficial part of the proximal coracoid underwent the greatest amount of osteolysis, whereas the distal portions, which are loaded by the conjoined tendon, demonstrated the least osteolysis and best bone healing. These findings were corroborated by the present study, which found that when severe osteolysis occurred, the proximal screw was always affected.

The present study did not identify an association between the presence of severe osteolysis and clinical outcomes. We propose, therefore, that asymptomatic coracoid graft osteolysis identified on postoperative imaging should not be reported as a complication following the Latarjet procedure.^3^^,^^5^^,^^6^^,^^12^ Rather, it is a natural phenomenon that occurs in accordance with Wolff’s Law that does not compromise clinical outcomes.

Although soft tissue stabilizations significantly decrease the likelihood of recurrent instability, up to 29% of these patients redislocate their shoulders within 4 years of surgery.^14^ A meta-analysis of 8 comparative cohort studies showed that the Latarjet conferred a significantly lower risk of recurrent instability.^2^ To demonstrate statistical significance despite a selection bias, where inclusion criteria for Latarjet groups would have included higher risk patients, underscores the efficacy of the Latarjet procedure.

Despite excellent clinical results, the Latarjet procedure is typically reserved for young, active patients with significant attritional bone loss.^18^ While 20%-25% of glenoid bone loss has typically been used as the threshold for critical bone loss, subsequent investigations have shown that 13.5%-15% glenoid bone loss significantly compromised patient-rated outcome scores to a level consistent with an unacceptable outcome, even in the absence of recurrent instability.^11^^,^^15^^,^^16^ The effectiveness of the Latarjet procedure and the expansion of the eligibility criteria for this operation may explain why there has been a 250% increase in the number of Latarjet procedures performed over the last decade, with a projected 214% increase in the number of procedures performed by 2030 in the United States alone.^1^

The strength of this study was that, to our knowledge, it was the largest in terms of sample size and had the longest radiological follow-up of any study to investigate coracoid graft osteolysis following the Latarjet procedure to date. Nonetheless, this study had several important limitations. This included the loss to follow-up. In addition, of the patients who responded to the questionnaire, only 39% had CT scans that were performed ≥ 12 months postoperatively, wherein coracoid graft osteolysis was analyzed for its association with clinical outcomes.

## Conclusion

Severe osteolysis of the coracoid graft at the level of the proximal screw occurred in 29% (18/62 patients with ≥ 12 months of radiological follow-up) of shoulders at median 12 months post-Latarjet. Severe coracoid graft osteolysis did not compromise the stability of the shoulder, and in asymptomatic patients, should not be considered as a complication, but rather the expected sequelae in conformity with Wolff’s Law. The Latarjet is a reliable treatment for anterior shoulder instability in experienced subspecialty practices.

## Disclaimers:

Funding: No funding was disclosed by the authors.

Conflicts of interest: The authors, their immediate families, and any research foundation with which they are affiliated did not receive any financial payments or other benefits from any commercial entity related to the subject of this article.
