# Supplementary material for: An assessment of the clinical relevance of coracoid graft osteolysis following the Latarjet procedure: a clinical and radiological review
Source: JSES Int. 2024 Mar 28;8(4):719–23. doi: 10.1016/j.jseint.2024.03.004 (PMC11258820; doi:10.1016/j.jseint.2024.03.004)
Supplement: Supplementary File S1 [file mmc1.docx]

**Supplement File 1**. Association between WOSI scores and collected variables. Analysis performed using Bivariate Spearman’s correlation tests.

| **Variable** | **More likely to have better WOSI if:** | **Spearman’s rho** | **P value** |
| --- | --- | --- | --- |
| Age | Older | 0.183 | **0.022** |
| Self-funded | Not self-funded | -0.158 | **0.049** |
| Preoperative glenoid bone loss | More preoperative glenoid bone loss | 0.235 | 0.065 |
| Maximum Zhu grade (grade at the level of the screw with more osteolysis) | Had more osteolysis | 0.187 | 0.099 |
| Minimum Zhu grade (grade at the level of the screw with less osteolysis) | Had more osteolysis | 0.168 | 0.148 |
| Complete (Grade 3) osteolysis around both screws | Did not have complete osteolysis around both screws | -0.155 | 0.180 |
| Private | Privately insured | 0.139 | 0.083 |
| Previous surgery | Did not have ipsilateral shoulder surgery pre-Latarjet | -0.097 | 0.228 |
| Athlete | Was not an elite athlete | -0.09 | 0.264 |
| Subsequent surgery | Did not have subsequent ipsilateral shoulder surgery post-Latarjet | -0.067 | 0.408 |
| Sum of Zhu grades (sum of grades around both screws) | Had more osteolysis | 0.056 | 0.63 |
| Complete (Grade 3) osteolysis around one or more screws | Had complete osteolysis around at least one screw | 0.046 | 0.696 |
| Complete (Grade 3) osteolysis around neither screw | Did not have complete osteolysis around either screw | -0.046 | 0.696 |
| Worker’s compensation | Did not have a worker's compensation claim | -0.042 | 0.604 |
| Public | Public hospital patient | 0.022 | 0.782 |
| Re-Dislocation | Did not re-dislocate ipsilateral shoulder post-Latarjet | -0.007 | 0.929 |
| Sex | Female | -0.004 | 0.964 |
